# Prevalence and risk factors of strabismus in children and adolescents in South Korea: Korea National Health and Nutrition Examination Survey, 2008–2011

**DOI:** 10.1371/journal.pone.0191857

**Published:** 2018-02-14

**Authors:** Kyung Eun Han, Seung-Hee Baek, Seung-Hyun Kim, Key Hwan Lim, Epidemiologic Survey Committee of the Korean Ophthalmological Society

**Affiliations:** 1 Department of Ophthalmology, Ewha Womans University College of Medicine, Mok-dong Hospital, Seoul, South Korea; 2 Department of Ophthalmology, Kim's Eye Hospital, Konyang University College of Medicine, Seoul, South Korea; 3 Department of Ophthalmology, Korea University College of Medicine, Seoul, South Korea; Moorfields Eye Hospital NHS Foundation Trust, UNITED KINGDOM

## Abstract

**Purpose:**

To evaluate the prevalence and risk factors associated with horizontal strabismus in children and adolescents in South Korea.

**Methods:**

A total of 5,935 children and adolescents 5–18 years of age who participated in the fourth and fifth Korean National Health and Nutrition Examination Survey (KNHANES IV-V) from July 2008 to December 2011 were evaluated and the prevalence of horizontal strabismus was estimated. Univariate and multivariate logistic regression analyses were conducted to determine the association between demographic, socioeconomic and clinical risk factors and clinically significant exodeviation (≥15 prism diopters [PD]) and esodeviation (≥10 PD).

**Results:**

Among 5,935 eligible subjects, 84 subjects had clinically significant exodeviation and 13 had clinically significant esodeviation. The overall prevalence of clinically significant horizontal strabismus was 1.6% (95% confidence interval [CI], 1.2–2.1): 1.3% (95% CI, 1.0–1.7) for clinically significant exodeviation and 0.3% (95% CI, 0.1–0.6) for clinically significant esodeviation. Clinically significant exodeviation was associated with amblyopia (adjusted odds ratio [aOR], 6.45; 95% CI, 2.14–19.44), family history of strabismus (aOR, 4.91; 95% CI, 1.71–14.08) and astigmatism ≥1.0 D (aOR, 1.84; 95% CI, 1.13–2.98). Clinically significant esodeviation was associated with hyperopia (aOR, 12.16; 95% CI, 1.31–113.04) and amblyopia (aOR, 4.70; 95% CI, 1.12–19.81). Other demographic, socioeconomic, and clinical variables were not associated with strabismus.

**Conclusion:**

This study provides data on the prevalence and independent risk factors for clinically significant exodeviation and esodeviation in a representative population of children and adolescents in South Korea.

## Introduction

Strabismus is a common ocular disorder in children; the prevalence of strabismus ranges from 0.8% to 5.65% [1–9]. Significant strabismus left untreated can result in decreased binocularity and amblyopia, and could eventually lead to psychosocial problems, including low self-confidence, depressive mood disorder, reduced inter-personal relationships, and reduced employment [10–14].

The cause of strabismus is not well established. Various factors have been postulated to be associated with strabismus: ocular factors such as hyperopia, myopia, astigmatism, anisometropia, and amblyopia, family history of strabismus and amblyopia, maternal factors such as smoking or alcohol use during pregnancy, and perinatal factors such as intrauterine growth retardation, prematurity, and low birth weight [1–3, 15–18]. Among the ocular factors, hyperopia and esotropia are highly associated [2, 3, 16, 19]. Hyperopia gradually decreases during school-age years [20, 21]; however, most population-based studies evaluating associations between potential risk factors and strabismus have been conducted only in children aged 7 years or less (Table 1) [1–3, 15, 16]. The prevalence of myopia in children and adolescents in South Korea ranges from 50% in aged 5–11 years to 78.8% in aged 12–18 years [22], which is higher than that in China (16.2% in aged 5–15 years) [23] and that in Japan (43.5% at 12-year-old and 66.0% at 17-year-old) [20]. The exodeviation:esodeviation ratio was 3:1 in aged 3–5 years children in South Korea [24], which was lower than that in China (4:1 in aged 5–15-year-old) [4] and similar with that in Japan (2.8:1 in aged 6–13 years) [25]. Ethnicity may influence the prevalence of strabismus and exodeviation:esodeviation ratio, however, the relationship between possible risk factors and strabismus in South Korean has not been evaluated. Therefore, we evaluated strabismus prevalence and identified associations between demographic, socioeconomic, and clinical risk factors for strabismus in South Korean children and adolescents.

**Table 1:** Review of population-based studies of risk factors associated with esodeviation and exodeviation in children.

	Whole population Exodeviation, n (%) Esodeviation, n (%)	Age (years)	Associations between variables and exodeviation	Associations between factors and esodeviation
SMS* (Australia) 2006 [2]	1,740 14 (0.8%) with exotropia 26 (1.5%) with esotropia	6	Myopia ≤-0.5D, hyperopia ≥+3.0 D, astigmatism ≥1 D, anisometropia ≥1 D, and amblyopia (all p<0.05)	
ALSPAC (UK) 2008 [1]	7,825 45 (0.6%) with exodeviation 211 (2.8%) with esodeviation	7	-	Family history of strabismus and amblyopia OR 2.39 (95% CI 1.27–3.20) Parental hypermetropia OR 1.62 (95% CI 1.09–2.44)
MEPEDS & BPEDS (USA) 2011 [16]	9,970 102 (1.0%) with exotropia 102 (1.0%) with esotropia	0.5–6	Female sex OR 1.62 (95% CI 1.08–2.42) Astigmatism 1.5-<2.5 D and ≥2.5D compared to <0.5 D: OR 2.49 and 5.88, respectively (95% CI 1.30–4.79 and 2.76–12.54, respectively) J0 anisometropia 0.25–<0.5 D and ≥0.5D compared to <0.25 D: OR 2.01 and 2.63, respectively (95% CI 1.25–3.22 and 1.26–5.49, respectively)	Hyperopia 2-<3 D, 3-<4 D, 4-<5 D, and ≥5 D compared to 0-<1 D: OR 6.38, 23.06, 59.81 and 122.24, respectively (95% CI 2.56–15.93, 9.56–55.61, 23.06–151.52, and 49.86–299.70, respectively) Child age 48–59 months and 60–72 months compared to 6–11 months: OR 7.94 and 9.40, respectively (95% CI 1.85–34.03 and 2.20–40.10, respectively) Anisometropia ≥1 D OR 2.03 (95% CI 1.10–3.73)
STARS* (Singapore) 2013 [15]	2,992 20 (0.7%) with exotropia 3 (0.1%) with esotropia	0.5–6	Sibling with strabismus: OR 41.20 (95% CI 9.03–188.00) Astigmatism ≥1.0 D: OR 4.19 (95% CI 1.20–14.65) Amblyopia: OR 12.85 (95% CI 2.32–71.27) Paternal education tertiary and secondary compared to those with none/primary school: OR 0.25 and 0.12, respectively, (95% CI 0.07–0.96 and 0.02–0.77, respectively)	
NPVP (China) 2015 [3]	5,831 270 (4.6%) with exotropia 45 (0.8%) with esotropia	3–6	Myopia -1-<0 D and <-1 D compared to 0-<1 D: OR 40.54 and 18.93, respectively (95% CI 13.16–124.86 and 5.25–68.22, respectively) Hyperopia 1-<2 D, 2–<3 D, 3–<4 D and 4–<5 D compared to 0-<1 D: OR 67.78, 23.13, 25.57 and 8.36, respectively (95% CI 40.80–112.60, 12.70–42.13, 9.97–65.59 and 1.71–40.97, respectively) Astigmatism 0.5-<1.0 D and <0 D compared to 0–0.5 D: OR 3.56 and 1.90, respectively (95% CI 1.51–8.40 and 1.17–3.10, respectively)	Hyperopia 2-<3 D, 3-<4 D, 4-<5 D and ≥5 D compared to 0-<1 D: OR 9.30, 9.28, 14.57 and 180.82, respectively (95% CI 2.63–32.96, 1.48–58.13, 2.32–91.65, and 36.37–898.89, respectively) Anisometropia 0.5-<1 D and ≥1D compared to <0.5 D: OR 3.15 and 7.41, respectively (95% CI 1.07–9.29 and 2.50–21.93, respectively)

SMS = Sydney Myopia Study, ALSPAC = Avon Longitudinal Study of Parents and Children, MEPEDS = Multi-Ethnic Pediatric Eye Disease Study, STARS = Strabismus, Amblyopia and Refractive Error in Singaporean Preschoolers Study, NPVP = Nanjing Pediatric Vision Project

D = diopters, PD = prism diopters, OR = odds ratio, CI = confidence interval, J0 = power in the vertical or horizontal meridian

* Separate association analyses of esodeviation and exodeviation were not reported.

## Methods

### Study population

The Division of Chronic Disease Surveillance, Korea Centers for Disease Control and Prevention, Ministry of Health and Welfare regularly conduct a nationwide, population-based, cross-sectional health examination and survey, the Korea National Health and Nutrition Examination Survey (KNHANES), to evaluate the general health and nutritional status of South Koreans. KNHANES consists of 3 parts, a health interview survey, a health examination survey, and a nutrition survey. The health interview data were collected *via* household interviews and health examination data were collected in a specially-equipped mobile examination center. To represent the South Korean population, this survey had a stratified, multistage probability sampling design based on National Census Data. Additional details of the KNHANES design and methods have been described previously [22].

KNHANES data were collected in 1998 (I), 2000 (II), 2005 (III), 2007–2009 (IV), 2010–2012 (V), and 2013–2015 (VI). From July 2008 to December 2011, KNHANES included an ophthalmologic survey and examination for subjects aged 3 years or older. Ocular alignment assessment was performed on all subjects, but visual acuity (VA) assessment and refraction using an autorefractor were performed on subjects aged 5 years or older. Full ophthalmologic examinations, including slit lamp examination and intraocular pressure measurements, were performed on subjects aged 19 years or older.

**Fig 1:**
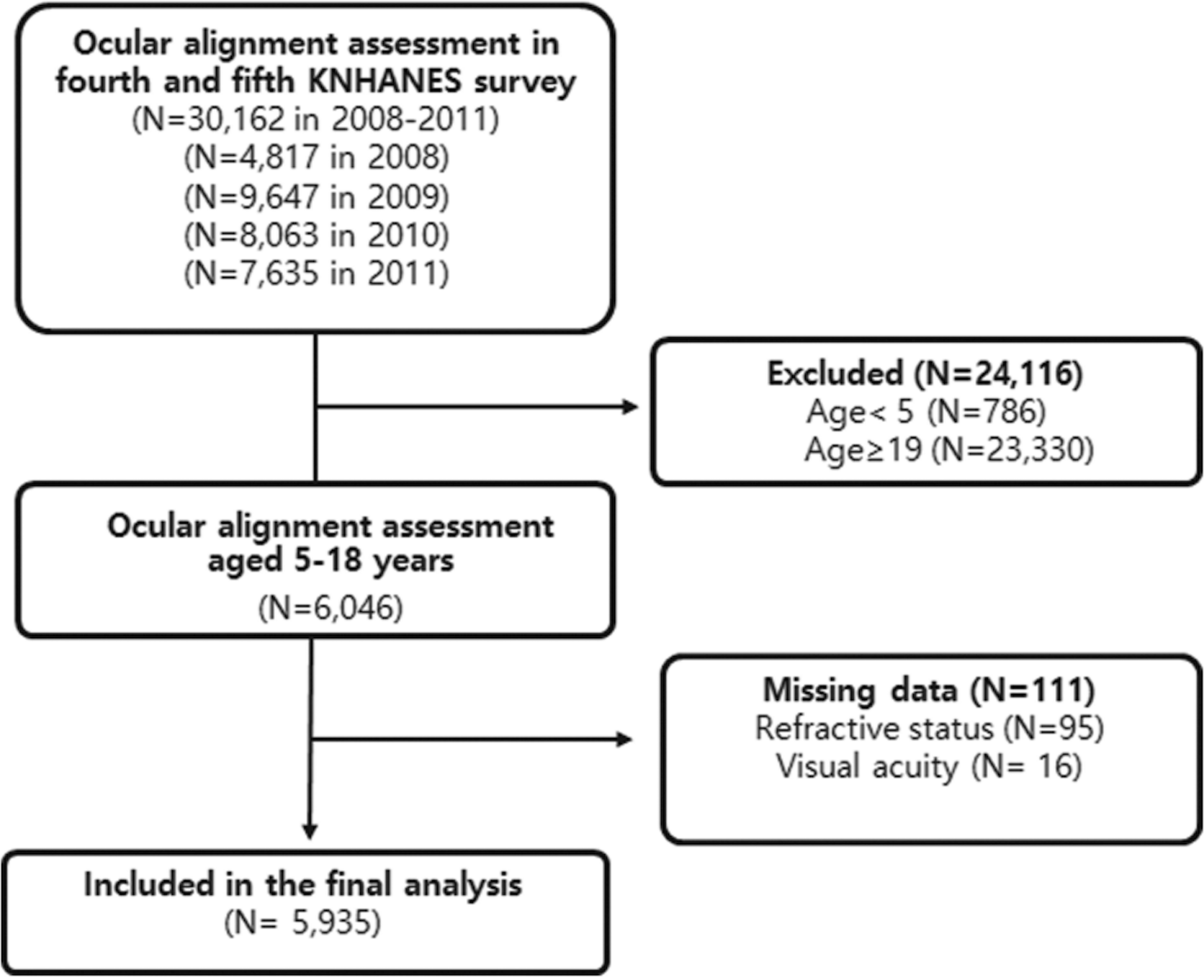
A flowchart showing study participants for final analysis.

Among the 30,401 subjects aged 3 years or older who underwent ophthalmic examination, 30,162 subjects underwent ocular alignment assessment. Of these, 24,227 subjects were excluded for the following reasons: 1) <5 years of age or >18 years of age, 2) missing refractive error data, or 3) missing VA assessment data (Fig 1). All missing data resulted from nonresponse. Finally, a total of 5,935 subjects (3,116 males and 2,819 females) were included in the present study.

This survey was reviewed and approved by the Institutional Review Board of the Korean Centers for Disease Control and Prevention and all participants provided written informed consent. The study was conducted in accordance with the Declaration of Helsinki.

### Questionnaire and demographic and socioeconomic variables

A detailed interviewer-administered questionnaire regarding socioeconomic and medical information was obtained from one of the subject’s parents or an accompanying guardian. All subjects were asked the following questions: “Have you ever been diagnosed with any disease by physicians?” If a subject answered yes, the subject was asked to record the specific disease name. “How much is whole income of your family?” The answer to this question was to write in exact amount and it was recorded into quartiles. “Do your family have your own house?” The answer would be yes or no, if yes, the subject had to choose one or more.

Demographic and socioeconomic data included the following variables: age (5–8 years, 9–12 years, 13–15 years, or 16–18 years), sex (male or female), residential area (urban or rural), monthly household income (lowest-second quartile or third to highest quartile), house ownership (yes or no), and highest maternal educational level achieved (middle school graduate or lower or high school graduate or higher).

### VA assessment and definitions for refractive error and amblyopia

With subjects wearing their glasses (if applicable), uncorrected VA and spectacle-corrected VA were evaluated from a distance of 4 m using an international standard vision chart based on the logarithm of the minimum angle of resolution (logMAR) scale (Jin’s vision chart, Seoul, South Korea). Refractive data of all participants were obtained using an autorefractor-keratometer (KR8800; Topcon, Tokyo, Japan). If the uncorrected or spectacle-corrected VA was lower than logMAR 0.1 (Snellen, 20/25), corrected VA was obtained after autorefraction. If the corrected VA after autorefraction was lower than logMAR 0.1, a pinhole was added to measure the final corrected VA.

Refractive errors were classified using the spherical equivalents (SE), which were calculated as the spherical value plus 1/2 cylindrical value. Emmetropia was defined as -0.5 to <+0.5 diopters (D); mild myopia was defined as -3.0 to <-0.5 D; moderate myopia was defined as -6.0 to <-3.0 D; high myopia was defined as <-6.0 D; and hyperopia was defined as ≥+0.50 D. If anisometropia was present, the refractive error was defined as SE of the less hyperopic eye because accommodative convergence is potentially driven by accommodation of the less hyperopic eye. Astigmatism was defined as a cylindrical error ≥1.0 D. If astigmatism was bilaterally present, the more astigmatic eye was used to measure astigmatic refractive error. Unilateral amblyopia was defined as at least a 2-line interocular difference in BCVA with <20/32 in the worse eye. Bilateral amblyopia was defined as decreased BCVA ≤20/40 in both eyes. Clinical variables included the following variables: refractive error (emmetropia, mild myopia, moderate myopia, high myopia, and hyperopia), anisometropia (<1.0 D or ≥1.0 D), and astigmatism (<1.0 D or ≥1.0 D).

### Ocular alignment assessment and strabismus definition

Ocular alignment was evaluated using the alternate prism and cover test with fixation targets at 4-m distance. Patients wore glasses (if applicable) and were evaluated by well-trained ophthalmology residents. When a subject’s visual acuity was insufficient to maintain fixation on a distant target, the corneal reflection test was used with or without a prism. Any abnormalities, including horizontal or vertical strabismus, were recorded. Among these, horizontal strabismus was categorized into one of the following: exodeviation ≥15 prism diopter (PD), exodeviation 1–14 PD, orthotropia, esodeviation 1–9 PD, and esodeviation ≥10 PD. We decided exodeviation ≥15 PD and esodeviation ≥10 PD were clinically significant strabismus, which may be a threshold for strabismus surgery [26–28]; thus, we used this classification for association analyses. A first-degree relative with a history of strabismus was also included.

### Statistical analysis

KNHANES sampling was weighted by adjusting for oversampling and nonresponses [29]. Statistical analyses were performed using SPSS software, version 23.0 (SPSS, Inc., Chicago, IL, USA) and SAS software, version 9.4 (SAS, Inc., Cary, NC, USA). Prevalence was assessed with a 95% confidence interval (CI). Univariate and multivariate logistic regression analyses were conducted to evaluate associations among age, sex, highest maternal education, residential area, house ownership, monthly income, refractive error, anisometropia, astigmatism, amblyopia, and family history of strabismus. Less hyperopic eye for SE refractive error and the more astigmatic eye for astigmatism were chosen for analyses. To measure the association between risk factors and horizontal strabismus, odds ratios (ORs) and 95% CIs were calculated. P values <0.05 were considered statistically significant.

## Results

### Prevalence of strabismus

Among the eligible 5,935 subjects, 5,275 subjects (88.9%) had orthotropia, 533 (8.9%) had an exodeviation 1–14 PD, 84 (1.4%) had an exodeviation ≥15 PD, 13 (0.2%) had an esodeviation ≥10 PD, and 30 (0.5%) had an esodeviation 1–9 PD. The estimated prevalence of all exodeviations was 10.5% (95% CI, 9.3–11.8), and the prevalence of all esodeviations was 0.8% (95% CI, 0.5–1.2). The overall estimated prevalence of clinically significant horizontal strabismus (exodeviation ≥15 PD or esodeviation ≥10 PD) was 1.6% (95% CI, 1.2–2.1). Among them, three subjects in clinically significant exodeviation had been diagnosed with strabismus and no one had been diagnosed with strabismus in clinically significant esodeviation.

### Associations of demographic, socioeconomic, and clinical factors with strabismus

General characteristics of the study population in association analyses are shown in Table 2.

**Table 2:** Clinical characteristics of subjects for association analysis (n = 5,935).

Variables	n	%	Clinically significant exodeviation	Clinically significant esodeviation
			(n = 84, 1.4%)	(n = 13, 0.2%)
Age				
5–8 years	1751	23.1	25	5
9–12 years	1858	28.7	27	3
13–15 years	1289	24	16	3
16–18 years	1037	24.1	16	2
Sex				
Female	2819	47	44	5
Male	3116	53	40	8
Maternal education				
Middle school or lower	450	9.1	8	1
High school or higher	4858	76.9	61	9
Residential area				
Rural	926	16.8	19	1
Town	5009	83.2	65	12
House ownership				
No	1997	36.9	23	6
Yes	3922	62.8	59	7
Monthly income				
Low	2154	41.1	33	5
High	3704	57.5	47	8
SE refractive error*				
-0.5 to < 0.5 D (emmetropia)	1629	27.4	17	1
≥ 0.5 D (hyperopia)	285	4.8	5	4
-3.0 to < -0.5 D (mild myopia)	2484	41.9	32	3
-6.0 to < -3.0 D (moderate myopia)	1179	19.9	25	4
< -6.0 D (high myopia)	358	6	5	1
Anisometropia				
< 1.0 D	5131	86.5	66	9
≥ 1.0 D	804	13.5	18	4
Astigmatism*				
< 1.0 D	3917	66	41	4
≥ 1.0 D	2018	34	43	9
Amblyopia				
No	5858	98.7	79	11
Yes	77	1.3	5	2
Family history of strabismus				
No/No response	5723	96.4	76	13
Yes	115	3.6	8	0

SE = spherical equivalent

*Less hyperopic eye for SE refractive error and the more astigmatic eye for astigmatism were chosen for analyses.

The associations between various risk factors and clinically significant exodeviation are shown in Table 3. After adjusting for all potential risk factors, amblyopia (adjusted odds ratio [aOR], 6.45; 95% CI, 2.14–19.44; p = 0.001), family history of strabismus (aOR, 4.91; 95% CI, 1.71–14.08; p = 0.003) and astigmatism ≥1.0 D (aOR, 1.84; 95% CI, 1.13–2.98; p = 0.014) were significantly associated with clinically significant exodeviation. Age group, sex, highest maternal education, residential area, house ownership, monthly income, refractive error and anisometropia were not associated with exodeviation in either univariate or multivariate logistic regression analyses.

**Table 3:** Univariate and multivariate logistic regression analysis for clinically significant exodeviation in children and adolescents.

	Univariate analysis			Multivariate analysis*		
Risk factors	OR	95% CI	P	aOR	95% CI	P
Age						
5–8 years	1.0 (ref)			1.0 (Ref)		
9–12 years	0.83	0.45–1.55	0.562	0.71	0.35–1.45	0.341
13–15 years	0.70	0.33–1.51	0.365	0.44	0.19–1.04	0.061
16–18 years	0.87	0.40–1.90	0.733	0.53	0.22–1.29	0.162
Sex						
Female	1.0 (ref)			1.0 (Ref)		
Male	0.96	0.57–1.63	0.892	0.93	0.56–1.57	0.792
Maternal education						
Middle school or lower	1.0 (Ref)			1.0 (Ref)		
High school or higher	0.51	0.21–1.24	0.139	0.51	0.23–1.11	0.083
Residential area						
Rural	1.0 (Ref)			1.0 (Ref)		
Town	0.62	0.32–1.20	0.156	0.62	0.35–1.11	0.109
House ownership						
No	1.0 (Ref)			1.0 (Ref)		
Yes	1.11	0.61–2.04	0.729	1.19	0.64–2.19	0.587
Monthly income						
Low	1.0 (Ref)			1.0 (Ref)		
High	0.76	0.44–1.31	0.314	0.84	0.50–1.38	0.484
SE refractive error†						
-0.5 to < 0.5 D (emmetropia)	1.0 (Ref)			1.0 (Ref)		
≥ 0.5 D (hyperopia)	2.54	0.65–9.93	0.179	1.69	0.56–5.11	0.352
-3.0 to < -0.5 D (mild myopia)	1.20	0.57–2.50	0.637	1.20	0.57–2.51	0.631
-6.0 to < -3.0 D (mod myopia)	1.93	0.91–4.13	0.088	1.94	0.86–4.39	0.110
< -6.0 D (high myopia)	1.37	0.45–4.16	0.575	1.33	0.39–4.51	0.647
Anisometropia						
< 1.0 D	1.0 (Ref)			1.0 (Ref)		
≥ 1.0 D	1.38	0.76–2.51	0.287	1.01	0.52–1.96	0.973
Astigmatism†						
< 1.0 D	1.0 (Ref)			1.0 (Ref)		
≥ 1.0 D	**2.00**	**1.19–3.37**	**0.009**	**1.84**	**1.13–2.98**	**0.014**
Amblyopia						
No	1.0 (Ref)			1.0 (Ref)		
Yes	**7.92**	**2.24–28.02**	**0.001**	**6.45**	**2.14–19.44**	**0.001**
Family history of strabismus						
No/No Response	1.0 (Ref)			1.0 (Ref)		
Yes	**4.49**	**1.65–12.23**	**0.003**	**4.91**	**1.71–14.08**	**0.003**

OR = odds ratio, aOR = adjusted odds ratio, CI = confidence interval, SE = spherical equivalent refractive error, D = diopters, mod = moderate

Odds ratios in boldface are statistically significant.

*Adjusted for all variables listed in the table.

† Less hyperopic eye for SE refractive error and the more astigmatic eye for astigmatism were chosen for analyses.

The associations between various risk factors and clinically significant esodeviation are shown in Table 4. Hyperopia (aOR, 12.16; 95% CI, 1.31–113.04; p = 0.028) and amblyopia (aOR, 4.70; 95% CI, 1.12–19.81; p = 0.035) were associated with clinically significant esodeviation after adjusting for other variables. Other variables were not associated with clinically significant esodeviation in either univariate or multivariate logistic regression analyses.

**Table 4:** Univariate and multivariate logistic regression analysis for clinically significant esodeviation in children and adolescents.

	Univariate analysis			Multivariate analysis*		
Risk factors	OR	95% CI	P	aOR	95% CI	P
Age						
5–8 years	1.0 (ref)			1.0 (Ref)		
9–12 years	1.32	0.25–6.97	0.742	1.11	0.30–4.07	0.880
13–15 years	0.86	0.14–5.30	0.866	0.55	0.09–3.46	0.523
16–18 years	0.84	0.14–5.03	0.849	0.52	0.06–4.57	0.550
Sex						
Female	1.0 (ref)			1.0 (Ref)		
Male	2.23	0.69–7.20	0.180	2.28	0.68–7.65	0.181
Maternal education						
Middle school or lower	1.0 (Ref)			1.0 (Ref)		
High school or higher	1.40	0.16–12.11	0.759	1.05	0.10–10.96	0.966
Residential area						
Rural	1.0 (Ref)			1.0 (Ref)		
Town	3.45	0.42–28.35	0.249	3.01	0.35–26.91	0.311
House ownership						
No	1.0 (Ref)			1.0 (Ref)		
Yes	1.38	0.39–4.89	0.621	1.61	0.42–6.20	0.486
Monthly income						
Low	1.0 (Ref)			1.0 (Ref)		
High	1.19	0.30–4.71	0.803	1.02	0.37–2.83	0.967
SE refractive error†						
-0.5 to < 0.5 D (emmetropia)	1.0 (Ref)			1.0 (Ref)		
≥ 0.5 D (hyperopia)	**19.57**	**2.13–179.79**	**0.009**	**12.16**	**1.31–113.04**	**0.028**
-3.0 to < -0.5 D (mild myopia)	2.77	0.27–28.74	0.392	2.31	0.19–28.19	0.513
-6.0 to < -3.0 D (mod myopia)	10.04	0.97–104.24	0.053	6.45	0.55–75.67	0.138
< -6.0 D (high myopia)	3.20	0.20–51.70	0.411	1.62	0.08–33.77	0.754
Anisometropia						
< 1.0 D	1.0 (Ref)			1.0 (Ref)		
≥ 1.0 D	4.24	0.96–18.78	0.058	2.74	0.76–9.88	0.124
Astigmatism†						
< 1.0 D	1.0 (Ref)			1.0 (Ref)		
≥ 1.0 D	3.70	1.00–13.74	0.051	2.32	0.63–8.54	0.206
Amblyopia						
No	1.0 (Ref)			1.0 (Ref)		
Yes	**10.47**	**2.13–51.60**	**0.004**	**4.70**	**1.12–19.81**	**0.035**

OR = odds ratio, aOR = adjusted odds ratio, CI = confidence interval, SE = spherical equivalent refractive error, mod = moderate, D = diopter

Odds ratios in boldface are statistically significant.

*Adjusted for all variables listed in the table.

† Less hyperopic eye for SE refractive error and the more astigmatic eye for astigmatism were chosen for analyses.

## Discussion

Previous population-based studies reported varying strabismus prevalence based on the age and ethnicity of the population or the study design; strabismus prevalence in children has been reported to be from 0.8% (Singapore) to 5.65% (China) [1–9]. Population-based studies regarding the prevalence of strabismus in South Korea are limited; only one study reported the prevalence of manifest strabismus (the definition and classification of strabismus were not disclosed) as 5.8% in 36,973 kindergarten children 3–5 years of age [30]. In the present study, the estimated prevalence of clinically significant horizontal strabismus was 1.6%, which is lower than that of previous studies conducted in South Korea, China, USA, and UK [1, 5, 7–9, 30, 31], similar to Japan [32], and higher than Singapore [6]. Because this study evaluated the prevalence of “clinically significant” horizontal strabismus, a direct comparison of our results with those of other studies that evaluated only manifest strabismus is not appropriate. Only Avon Longitudinal Study of Parents and Children (ALSPAC) took into account the prevalence of latent strabismus and found the prevalence of clinically significant divergence (manifest or latent with ≥15 PD) was 0.6% (95% CI; 0.5–0.8) and clinically significant convergence (manifest or latent with ≥10 PD) was 2.8% (95% CI; 2.5–3.3) in UK children [1].

The exodeviation:esodeviation ratio was 6.4:1, and the higher proportion of exodeviation was similar to other studies of Asian populations (ratio range: 2.5:1 to 51.0:1) [5, 6, 8, 32]. This may correlate with lower hyperopia prevalence in Korea compared with other Asian populations. In contrast, Caucasian populations who have a higher prevalence of hyperopia showed a similar or inverse exodeviation:esodeviation ratio [1, 9, 16, 17].

Strabismus presence was significantly associated with several variables. Among the various potential risk factors, amblyopia was the only factor that influenced both strabismus types; subjects with amblyopia were 6.45 times more likely to have exodeviation and 4.70 times more likely to have esodeviation. In the Strabismus, Amblyopia and Refractive Error in Singaporean Preschoolers Study (STARS), children with strabismus showed a 12.85-fold association with amblyopia [15]. The Sydney Myopia Study (SMS) also reported that children with strabismus were statistically significantly more likely to have amblyopia than children without strabismus (p < 0.001) [2]. The present study clearly showed an association between amblyopia and both strabismus types.

Refractive error and strabismus are closely related [2, 15, 17, 33]. Among refractive error types, associations between hyperopia and esotropia have been firmly established, especially in children who have significant hyperopia (≥+3.00 D) [2, 16, 19]. However, myopia and exotropia, both more prevalent in Asian subjects, have not been thoroughly evaluated. Consistent with previous studies of Asian populations [34–36], myopia was more prevalent than hyperopia in our study; 67.8% of subjects were myopic, 27.4% of subjects were emmetropic and only 4.8% of subjects were hyperopic. To explore the relationship between myopia and exodeviation in detail, we divided myopia into degrees based on SE level: mild (-3.0 to <-0.5 D), moderate (-6.0 to <-3.0 D) and high (<-6.0 D). However, we found no association between any myopia level and clinically significant exodeviation. Previous studies have reported conflicting results. The STARS, another population-based study of Asian children, found that refractive error was not an independent risk factor for strabismus [15]. Meanwhile, subgroup analyses in the Multi-Ethnic Pediatric Eye Disease Study (MEPEDS) showed that subjects with myopia ≤-1.0 D in at least 1 eye were 2.46 times more at risk for exotropia compared with subjects with emmetropia <1.0 D [16]. SMS reported that all refractive errors, including myopia, significant hyperopia (≥+3.0D), astigmatism, and anisometropia, were more common in children with strabismus [2]; however, exodeviations and esodeviations were not separately analyzed. The Nanjing Pediatric Vision Project (NPVP) reported that Chinese children with mild myopia <-1.0 D had a 40-fold greater risk of exotropia compared with mild hyperopia 0 to <1.0 D [3]. However, among the 5,831 Chinese children in the NPVP [3], myopia was only found in 44 (0.8%) children and the myopia rate was lower than in our study population. Increased accommodative demand in exodeviation could account for this association [37–39]. Further research is needed to clarify the relationship between exodeviation and myopia.

Hyperopia was found to increase the risk of esodeviation in the present study. Several studies have shown that hyperopia is risk factor for esotropia [1–3, 9, 15] and the risk increases with each D of increasing hyperopia [3, 16]. The risk of esotropia in children with hyperopia ≥+5.0 D was 180 times greater than in children with <+1.0 D of hyperopia [3]. However, in the present study, due to the low proportion of high hyperopia subjects in our study population, further analyses based on levels of hyperopia were not feasible.

Astigmatism ≥1.0 D was associated with a 1.84-fold increased risk for clinically significant exodeviation in this study. In line with this study result, STARS reported astigmatism ≥1.0 D increased the risk of strabismus (the direction was not mentioned) 4 times more than astigmatism <1.0 D.^7^ In the MEPEDS and Baltimore Pediatric Eye Disease Study (BPEDS), astigmatism ≥2.5 D was associated with 6-fold risk of exotropia in 6- to 72-month old children [16]. In the NPVP, hyperopic astigmatism 0.5 to <1.0 D and all myopic astigmatisms were independent risk factors for exotropia (aOR, 3.56 and 1.90, respectively) [3]. The different influences of astigmatism on exodeviation and esodeviation require additional research.

Although no specific genetic loci for strabismus have been determined, several studies concluded it is heritable [19]. A study regarding associations between siblings and strabismus from the Collaborative Perinatal Project reported that any sibling pair had more than twice the risk of developing exotropia or esotropia compared with siblings from separate single births, respectively [40]. STARS also reported that a child who had a sibling with strabismus was 41 times more likely to develop strabismus (95% CI, 9.03–188.00) [15]. ALSPAC showed that a family history of strabismus/amblyopia was associated with convergent strabismus (aOR, 1.38; 95% CI, 0.94–2.03), but not with divergent strabismus [1]. MEPEDS and BPEDS reported that a family history of strabismus was associated with 2-fold increased risk of exotropia (OR, 2.29; 95% CI, 1.24–4.13; p = 0.006) [16]. In this study, subjects who had a family history of strabismus were 4.82 times more likely to develop exodeviation than subjects without a family history. None of the children with esodeviation in our study had family histories of strabismus; however, our study included only a few children with esodeviation, thus, drawing conclusions from this sub-sample is difficult.

Associations between socioeconomic variables and pediatric strabismus yielded conflicting results. STARS reported that higher paternal education had a protective effect against strabismus and in the Millennium Cohort Study of UK children, socioeconomic status was inversely associated with strabismus [31]; however, SMS, MEPED, BPEDS, and ALSPAC reported that parental education, monthly income, and house ownership were not associated with strabismus [1, 15, 16]. We also did not find any associations between maternal education, monthly income, house ownership, or residential area and clinically significant strabismus, similar to these previous studies.

This study has several limitations. Refractive errors were not evaluated under cycloplegic conditions, which could bias the results in younger subjects who tended to accommodate more actively than older subjects. Studies of refractive errors measured under non-cycloplegic conditions found a myopic shift in 0.19–1.19 D when compared with cycloplegic refraction [41–44], but, hyperopic shifts may exist in some subjects [43]. Hence, we set the reference level for emmetropia to -0.5 D to <+0.5 D. However, this study results should be interpreted with a caution considering the non-cycloplegic nature of refraction measurements. Second, the health survey stage lacked clinical detail and did not yield reliable data on heterophoria and heterotropia. A certain amount of heterophoria is considered physiological and a small angle in horizontal heterotropia cases is neither cosmetically noticeable nor likely to lead to asthenopia [45]. Therefore, only significant degrees of ocular deviation were considered to be detected reliably and were considered for the present analysis. Third, ocular alignment assessment was carried out by multiple trainee ophthalmologists, but the KNHANES conducted quality management continuously to make up for the weaknesses. Forth, because slit lamp examination was not performed on our subjects, other potential risk factors associated with intraocular structures were not evaluated in this study. Finally, perinatal factors such as birth weight, gestational age and maternal age at birth were only documented in around half of children, and we therefore did not evaluate the association of these factors with strabismus.

In spite of these limitations, this study is a large population-based survey analyzed using a stratified and multistage probability sampling design. The results are intended representative of the entire South Korean population, which is relatively homogenous, in terms of environment and ethnicity. A further highlight of the study is the detailed finding of an association between myopia level and exotropia, both more common than hyperopia and esotropia in Asian populations.
